# Ultrafast Coherent Phonon Dynamics in GeTe and GeSe: Contrasting Metavalent and Covalent Bonding

**DOI:** 10.1002/smll.76481

**Published:** 2026-10-27

**Authors:** Timo Veslin, Felix Hoff, Felix Nöhl, Elias Hildebrand, Jan Köttgen, Pierre Lucas, Matthias Wuttig

**Affiliations:** ^1^ Institute of Physics IA RWTH Aachen University Aachen Germany; ^2^ Department of Materials Science and Engineering University of Arizona Tucson Arizona USA; ^3^ Peter‐Grünberg‐Institute – JARA‐Institute Energy Efficient Information Technology (PGI‐10) Wilhelm‐Johnen‐Straße Jülich Germany

**Keywords:** anharmonicity, chalcogenides, coherent phonons, metavalent bonding, phase change materials, unconventional glasses

## Abstract

Phase‑change materials exhibit pronounced changes in optical and electronic properties between their amorphous and crystalline phases, which are closely related to differences in chemical bonding. Here, we investigate the ultrafast lattice dynamics of GeTe and GeSe thin films using femtosecond pump‐probe transient reflectivity combined with Raman spectroscopy and structural characterization. By comparing amorphous and crystalline phases of both materials, we explore how different bonding mechanisms influence coherent phonon generation following optical excitation. Crystalline GeTe exhibits strong coherent phonon oscillations associated with the *A*_1_ optical mode, pronounced fluence‑dependent phonon softening, and very short coherent phonon dephasing times, indicating a highly anharmonic and excitation‐sensitive lattice potential. In contrast, amorphous GeTe shows weaker oscillations and only minor fluence dependence. For GeSe, coherent phonons are weak in the crystalline phase and absent in the amorphous phase. Structural relaxation of the amorphous films below the glass transition temperature does not significantly affect the ultrafast response. These observations can be understood in terms of their distinct bonding mechanisms. While GeSe remains covalently bonded in both phases, crystalline GeTe exhibits metavalent bonding that is highly susceptible to electronic excitation. Our findings demonstrate that coherent phonon spectroscopy provides a sensitive probe of bonding in chalcogenide materials.

## Graphical abstract

Ultrafast laser measurements reveal the differences in the optical response of atomic vibrations in GeTe and GeSe thin films. While crystalline GeTe exhibits strong, highly excitation‐sensitive coherent vibrations linked to metavalent bonding, covalently bonded GeSe shows weak or absent coherent phonons. This study demonstrates that ultrafast phonon spectroscopy can help distinguish bonding mechanisms in phase‐change materials with great sensitivity.

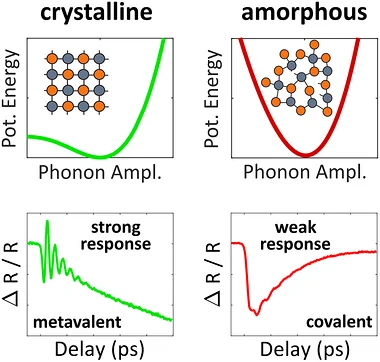

## 1 Introduction

Germanium telluride (GeTe) and germanium selenide (GeSe) are closely related chalcogenide compounds that exhibit markedly different physical properties despite being isoelectronic. GeTe is a prototypical phase‐change material (PCM) characterized by a large contrast in optical and electrical properties between its amorphous and crystalline phases and by its ability to undergo rapid and reversible structural transformations [1, 2]. These characteristics have enabled applications ranging from non‐volatile memory devices to tunable photonic components [2, 3, 4, 5, 6]. In contrast, GeSe is commonly described as a predominantly covalent chalcogenide semiconductor whose amorphous and crystalline phases exhibit more moderate property differences and comparatively low optical losses [7, 8, 9]. Layered and few‐layer GeSe have also attracted increasing interest as anisotropic two‐dimensional semiconductors for optoelectronic and ferroelectric applications [10, 11].

A particularly striking feature of GeTe is the pronounced change in optical reflectivity between its amorphous and crystalline phases [12, 13]. This contrast significantly exceeds what would be expected from density changes alone as described by the Clausius‐Mossotti relation, which relates variations in refractive index to changes in material density [14]. The unusually large optical contrast has therefore been attributed to substantial changes in chemical bonding upon crystallization [8, 12, 15]. While the amorphous phase is generally described as covalently bonded, the crystalline phase has been proposed to exhibit metavalent bonding characterized by enhanced electronic delocalization and large lattice polarizability [8, 16].

In contrast, GeSe is widely regarded as a covalent system in both its amorphous and crystalline phases [17, 18, 19]. Consequently, the optical contrast between these phases is largely governed by structural density changes and remains broadly consistent with the expectations of the Clausius‐Mossotti relation [14, 20]. The differing optical responses of GeTe and GeSe therefore provide a useful platform for exploring how variations in chemical bonding influence the physical properties of chalcogenide materials.

Crystalline GeSe is structurally related to black phosphorus and adopts an orthorhombic layered structure with strong covalent bonding within the layers and comparatively weak interlayer interactions [10]. Crystalline GeTe, by contrast, is commonly described as a Peierls‐distorted rocksalt structure with alternating short and long Ge‐Te bonds along the [111] direction [21]. This distortion gives rise to a bilayer‐like structural motif. However, the coupling between adjacent bilayers remains much stronger than the van der Waals‐like interlayer coupling in GeSe.

While the equilibrium optical and electronic properties of GeTe and GeSe have been studied extensively [7, 12, 13, 22], considerably less is known about how their lattice dynamics respond to strong optical excitation on ultrafast time scales. Femtosecond pump‐probe spectroscopy provides a powerful tool to investigate these dynamics [23, 24]. Following excitation by an ultrashort laser pulse, rapid changes in carrier density and electronic temperature modify the interatomic potential and can launch coherent optical phonons [25, 26]. Since the shape and anharmonicity of this potential depend sensitively on the underlying bonding mechanism, coherent phonon dynamics provide a sensitive probe of the coupling between electronic excitation and lattice motion [27].

Vibrational properties of phase‐change materials have been investigated using Raman spectroscopy, which reveals characteristic phonon modes in both amorphous and crystalline chalcogenides [13, 28, 29, 30]. In addition, femtosecond pump‐probe studies have demonstrated the generation of coherent optical phonons in several phase‐change materials, most notably in crystalline GeTe and in Ge‐Sb‐Te alloys such as Ge_2_Sb_2_Te_5_ [31, 32, 33]. These studies have shown that coherent lattice vibrations provide valuable insight into electron‐phonon coupling and the anharmonic lattice potential. However, most ultrafast investigations have focused on materials with strong optical contrasts associated with phase‐change behavior, while comparative studies involving more covalently bonded chalcogenides remain scarce. In particular, the coherent phonon response of GeSe and its evolution across different amorphous structural states has not been systematically explored.

These considerations raise the question of how coherent lattice dynamics evolve across the amorphous‐crystalline phase transition and how they are influenced by the substitution of tellurium with selenium. Comparing GeTe and GeSe therefore provides a useful platform to explore how different bonding regimes affect coherent phonon dynamics in chalcogenide materials.

In this work, we investigate ultrafast lattice dynamics in amorphous and crystalline GeTe and GeSe thin films using femtosecond pump–probe transient reflectivity spectroscopy. To probe possible structural variations within the amorphous phase, we additionally prepared relaxed glass states by annealing below the glass transition temperature *T*_g_. The structural and vibrational properties of these phases were characterized by X‐ray diffraction (XRD) and Raman spectroscopy. By comparing the coherent phonon response across the different structural states, we clarify how bonding and structural order influence the generation and decay of coherent lattice vibrations following ultrafast optical excitation.

## 2 Results

### 2.1 Structural Characterization

The structural phases of the deposited films were verified using grazing‐incidence X‐ray diffraction (GIXRD). Figure 1 shows the normalized diffraction patterns of GeTe and GeSe thin films in the as‐deposited amorphous state, a relaxed amorphous state obtained by annealing below the glass transition temperature, and the crystalline phase.

**FIGURE 1:**
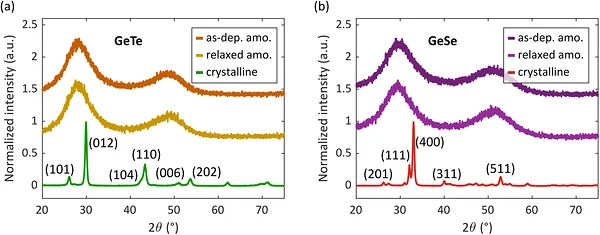
Grazing‐incidence X‐ray diffraction patterns of GeTe and GeSe thin films in three structural states. (a) GeTe in the as‐deposited amorphous, relaxed amorphous, and crystalline phases. The amorphous films exhibit diffuse scattering halos, while the crystalline film shows Bragg reflections characteristic of rhombohedral GeTe. (b) Corresponding diffraction patterns for GeSe, showing the transition from amorphous to crystalline orthorhombic GeSe upon annealing.

The as‐deposited GeTe film exhibits a broad diffuse scattering feature without distinct Bragg reflections, characteristic of an amorphous structure (Figure 1a). Structural relaxation of the amorphous phase was induced by annealing the films for 1 h at 145°C, a temperature chosen based on differential scanning calorimetry studies of sputtered chalcogenide glasses where structural relaxation occurs without crystallization [34, 35]. After crystallization at 300°C, sharp diffraction peaks appear that can be indexed to rhombohedral GeTe, confirming the formation of the crystalline phase [36].

A similar behavior is observed for GeSe (Figure 1b). The as‐deposited film displays a broad amorphous halo, while annealing for 1 h at 265°C results in a relaxed amorphous state without detectable crystalline reflections. Subsequent annealing at 350°C leads to the emergence of well‐defined diffraction peaks consistent with orthorhombic GeSe [37].

The absence of Bragg reflections after the sub‐*T*_g_ annealing step confirms that the relaxed films remain amorphous. These measurements therefore establish three well‐defined structural states, as‐deposited amorphous, relaxed amorphous, and crystalline, for both materials, which are used throughout the Raman and ultrafast pump–probe measurements discussed below.

### 2.2 Raman Spectroscopy

The vibrational properties of the films were investigated using Raman spectroscopy. Figure 2 shows the Raman spectra of GeTe and GeSe thin films for the three structural states introduced above: the as‐deposited amorphous state, the relaxed amorphous state obtained by sub‐*T*_g_ annealing, and the crystalline phase.

**FIGURE 2:**
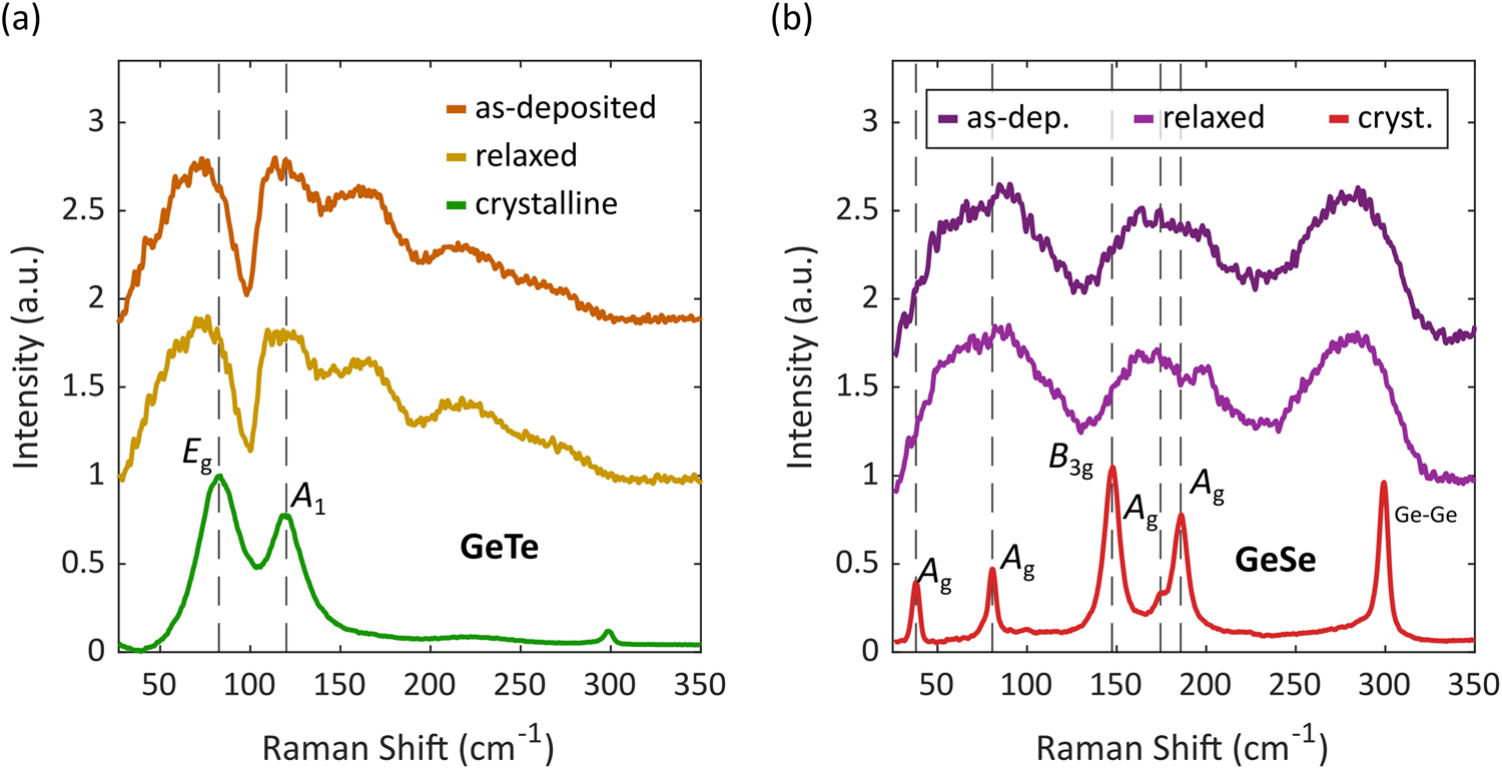
Raman spectra of GeTe and GeSe thin films in different structural states. (a) Raman spectra of GeTe in the as‐deposited amorphous state, a relaxed amorphous state obtained by annealing below the glass transition temperature, and the crystalline phase. (b) Corresponding spectra of GeSe for the same preparation conditions. Spectra are normalized and vertically offset for clarity.

The Raman spectra of the crystalline films exhibit well‐defined vibrational modes characteristic of the respective crystal structures. In contrast, the amorphous films display broad spectral features that reflect the structural disorder and the distribution of local bonding environments. These features are particularly weak in amorphous GeSe, where no well‐defined Raman peaks are observed. Upon crystallization, the spectra develop sharper and more clearly resolved peaks, consistent with the increased structural order of the crystalline lattice. We note that the spectra in Figure 2 are normalized and vertically offset to compare spectral shapes. The corresponding unnormalized spectra show that the Raman intensity of the amorphous films is much weaker than that of the crystalline films (Figure S8).

The Raman spectra of the as‐deposited and relaxed amorphous films are very similar for both GeTe and GeSe. Only minor changes in peak width and position are observed, indicating that annealing below the glass transition temperature mainly results in subtle structural relaxation rather than major modifications of the amorphous network.

The vibrational spectra of the two materials differ in accordance with their distinct crystal structures. Crystalline GeTe exhibits the characteristic *E*_g_ and *A*_1_ Raman modes [13], while crystalline GeSe displays several Raman‐active modes of *A*_g_ and *B*_3g_ symmetry [30]. A feature near 300 cm^−1^ is visible in the crystalline spectra and is commonly attributed to Ge‐Ge vibrations, which may indicate minor Ge segregation during crystallization [38, 39]. Overall, the Raman measurements establish the vibrational fingerprints of the crystalline phases and provide a reference for the phonon modes analyzed in the ultrafast pump‐probe experiments discussed below.

### 2.3 Ultrafast Transient Reflectivity

Figure 3 shows the transient reflectivity Δ*R*/*R* of the GeTe and GeSe films following femtosecond optical excitation at a representative incident pump fluence of approximately 1 mJ cm^−2^.

**FIGURE 3:**
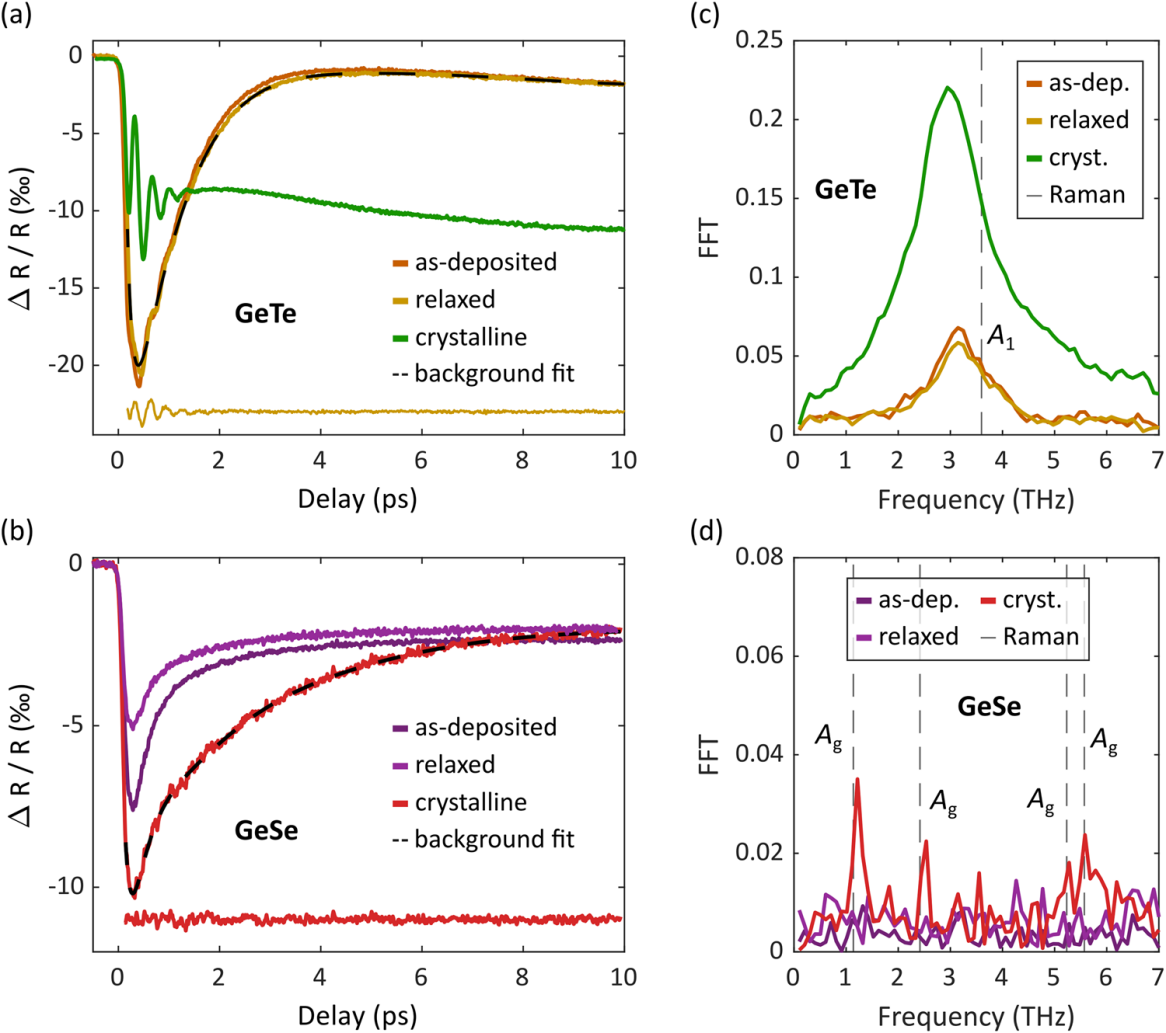
Transient reflectivity ΔR/R of GeTe and GeSe thin films measured by femtosecond pump‐probe spectroscopy at a representative pump fluence of approximately 1 mJ cm^−2^. (a) GeTe films in the as‐deposited amorphous, relaxed amorphous, and crystalline states. The crystalline film exhibits pronounced oscillations corresponding to the coherent *A*_1_ phonon, whereas the amorphous states show significantly weaker oscillatory contributions. The dashed line indicates the fitted electronic background. The oscillatory component of the relaxed amorphous sample obtained after background subtraction is shown below the traces with a vertical offset, revealing weak coherent oscillations that persist slightly longer than those observed in the crystalline phase. (b) Corresponding measurements for GeSe. The crystalline film shows only weak coherent oscillations, highlighted by the background‐subtracted component shown below the traces with a vertical offset, whereas no coherent oscillations are observed in the amorphous states. (c) Fourier transform spectra of the GeTe oscillatory components. A pronounced peak corresponding to the softened coherent A_1_ phonon is observed in the crystalline film, while the amorphous states exhibit much weaker spectral features. The dashed line indicates the Raman frequency of the *A*_1_ mode. (d) Corresponding Fourier transform spectra for GeSe. Several weak peaks in the crystalline phase coincide with Raman‐active phonon frequencies, while no distinct peaks are observed for the amorphous films.

The crystalline GeTe film exhibits the characteristic response reported for this material, i.e., a rapid initial decrease in reflectivity followed by pronounced oscillations arising from the coherent excitation of the *A*_1_ optical phonon (Figure 3a) [31]. The initial reflectivity change originates from the rapid excitation of charge carriers and the resulting modification of the dielectric function, forming the non‐oscillatory electronic background on which the coherent phonon oscillations are superimposed. The oscillation amplitude in crystalline GeTe is significantly larger than in the amorphous phases and decays within a few picoseconds due to strong dephasing of the coherent phonon. To highlight the weak oscillatory component in the amorphous films, the electronic background was fitted and subtracted from the transient reflectivity signal of the relaxed amorphous sample. The resulting oscillatory component is shown below the traces in Figure 3a. At longer delay times the reflectivity of the crystalline sample evolves slowly without fully recovering within the measured time window, indicating that the lattice remains out of equilibrium on this time scale, a behavior that is not observed for the amorphous films.

The two amorphous GeTe states display very similar transient responses. In particular, the overall decay envelope of the transient reflectivity signal remains essentially unchanged after structural relaxation of the glass. This indicates that annealing below the glass transition temperature does not significantly affect the coherent phonon dynamics and the electronic background response of the amorphous phase. Although long‐range translational symmetry is absent in amorphous materials, coherent oscillations following ultrafast excitation have previously been reported and are generally interpreted as synchronized vibrations of local structural units rather than phonons with well‐defined wave vectors [33, 40].

The oscillatory components were analyzed using both damped harmonic oscillator fits (shown in Figure S1) and Fourier transform (FFT) analysis. The extracted coherent phonon frequency is approximately 3.1 THz for the amorphous films and 2.9 THz for crystalline GeTe. The reduced frequency observed in crystalline GeTe compared to the equilibrium Raman value (3.6 THz) indicates a pronounced softening of the *A*_1_ mode under optical excitation in crystalline GeTe [32]. This will indeed be shown in the following. The corresponding coherent phonon dephasing times are about 0.47 ps for the amorphous states and 0.36 ps for the crystalline film. Here, the dephasing time describes the decay of the coherently excited phonon oscillation and therefore represents an effective lifetime of the coherent phonon that includes both population decay and loss of phase coherence. The obtained values are consistent with previous ultrafast studies on GeTe [31].

The transient reflectivity of GeSe films is shown in Figure 3b. In crystalline GeSe only weak oscillatory modulations are observed on top of the electronic background. Damped harmonic oscillator fits (see Figure S1d) reveal several modes near 1.2, 2.4, and 5.6 THz, consistent with the Raman‐active *A*_g_ phonons identified in Figure 2. Their amplitudes are more than an order of magnitude smaller than those observed in crystalline GeTe.

In contrast, neither the as‐deposited nor the relaxed amorphous GeSe films exhibit detectable coherent oscillations. Both amorphous states show a purely electronic response consisting of an initial reflectivity decrease followed by a recovery toward a quasi‐steady background level. The absence of coherent oscillations is consistent with the Raman spectra of amorphous GeSe, which exhibit only weak and broadened vibrational features without well‐defined peaks.

The FFT spectra shown in Figure 3c,d confirm these observations. In crystalline GeTe a pronounced peak appears near 3 THz corresponding to the coherent. *A*_1_ optical phonon, while the amorphous films exhibit much weaker spectral features. For crystalline GeSe several weak peaks near 1.2, 2.4, and 5.6 THz coincide with Raman‐active *A*_g_ modes, whereas no distinct peaks are observed for the amorphous films.

### 2.4 Fluence‐Dependent Phonon Dynamics

To investigate the excitation‐dependent lattice response, the oscillatory components of the transient reflectivity signals were analyzed as a function of pump fluence. The extracted coherent phonon frequencies, oscillation amplitudes, and dephasing times are summarized in Figure 4.

**FIGURE 4:**
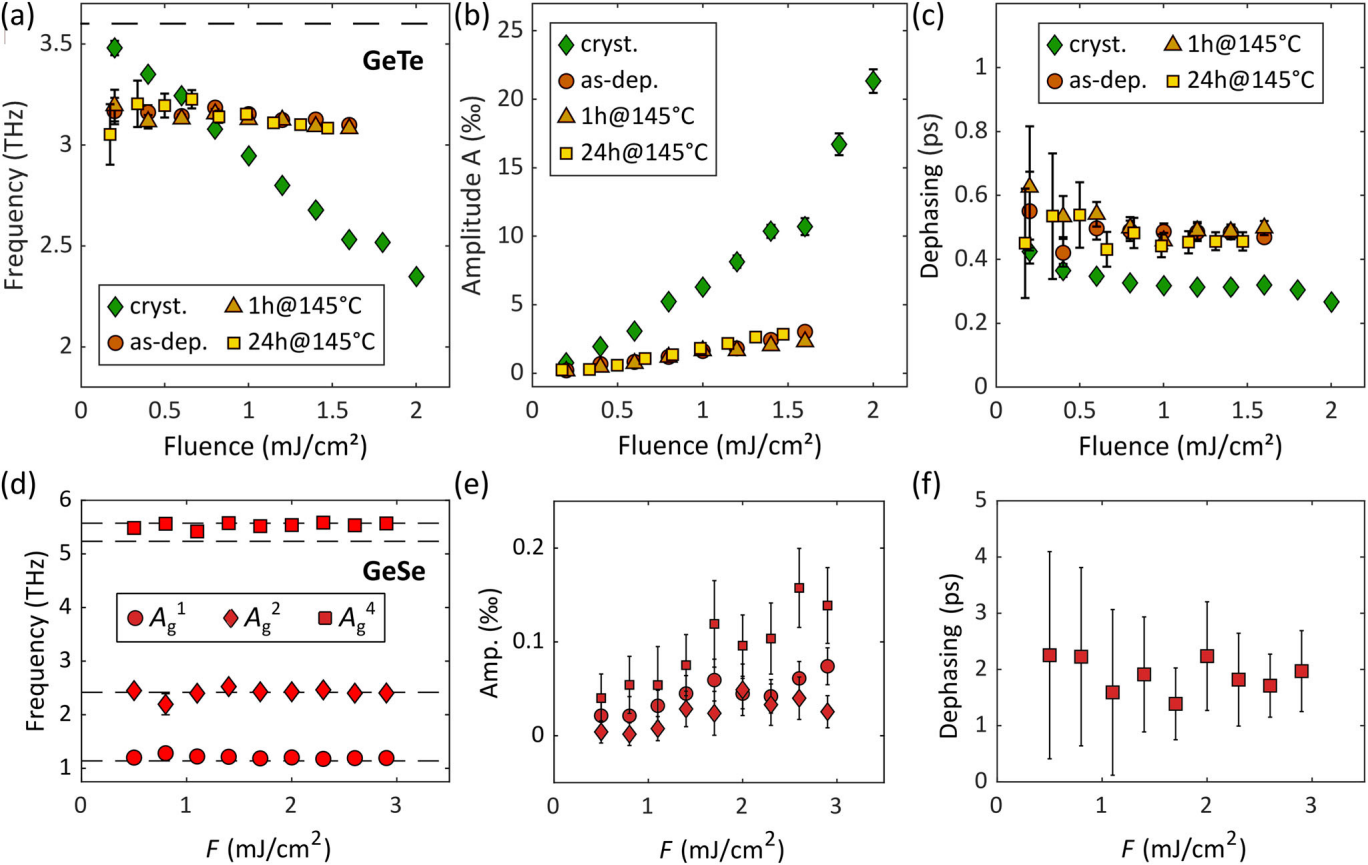
Fluence dependence of coherent phonon parameters extracted from damped harmonic oscillator fits. (a–c) GeTe films in the crystalline, as‑deposited amorphous, and relaxed amorphous states showing (a) phonon frequency, (b) oscillation amplitude, and (c) dephasing time as a function of incident pump fluence. The *A*_1_ phonon of the crystalline phase exhibits pronounced softening and strong amplitude growth with increasing fluence, while the amorphous phases show only weak fluence dependence. (d–f) Corresponding analysis for crystalline GeSe. The frequencies of the three coherent phonon modes assigned to Raman‐active *A*_*g*_ modes show only weak fluence‐dependent shifts, and both amplitude and dephasing time show only weak fluence dependence. Dashed lines indicate Raman frequencies obtained from equilibrium Raman spectroscopy.

For GeTe (Figure 4a–c), the crystalline phase exhibits a pronounced fluence dependence. With increasing pump fluence the frequency of the coherent *A*_1_ phonon decreases substantially, demonstrating strong photoinduced phonon softening, consistent with literature [31, 32, 41]. At the same time, the oscillation amplitude increases markedly over the investigated fluence range. In contrast, the amorphous GeTe films show only minor frequency shifts and a much weaker increase in oscillation amplitude. The as‐deposited and relaxed amorphous states display nearly identical behavior, indicating that structural relaxation below the glass transition temperature does not noticeably affect the excitation‐dependent phonon response. The extracted dephasing times remain on the sub‐picosecond scale for all GeTe samples, with the crystalline phase consistently exhibiting shorter dephasing times than the amorphous films despite its higher structural order.

A different behavior is observed for crystalline GeSe (Figure 4d–f). The frequencies of the three detected coherent phonon modes remain almost constant over the entire fluence range and agree well with the Raman frequencies obtained under equilibrium conditions. Quantitatively, the relative frequency shifts amount to approximately 1% or less per absorbed mJ cm^−2^, demonstrating that any photoinduced softening in GeSe is much weaker than the pronounced softening observed for crystalline GeTe. The oscillation amplitudes increase with fluence, although they remain more than an order of magnitude smaller than those observed in crystalline GeTe. For the $A_{g}^{4}$ mode, a linear fit yields an increase of the amplitude of approximately 0.41 × 10^−3^  in Δ*R*/*R* per absorbed mJ cm^−2^. The magnitude of the prompt reflectivity minimum likewise increases approximately linearly over the investigated fluence range, with no indication of saturation. Within experimental uncertainty, the extracted phonon dephasing times do not show a systematic fluence dependence. For clarity, only the dephasing time of the highest‐frequency mode is shown in Figure 4f, as the dephasing times of the lower‐frequency modes exceed the experimentally accessible time window and cannot be reliably determined.

To assess the effect of unequal optical excitation, we estimated the film absorption at the 800 nm pump wavelength from ellipsometric dielectric function data using a single‐pass, normal‐incidence approach described in Ref. [42]. The estimated absorbed fractions are 0.38 and 0.18 for crystalline GeTe and GeSe, respectively, and 0.59 and 0.39 for their amorphous counterparts. The maximum incident fluences correspond to approximately 0.75 mJ cm^−2^ absorbed in crystalline GeTe and 0.55 mJ cm^−2^ in crystalline GeSe. Thus, the absorbed‐fluence ranges overlap substantially, and the difference in absorption does not account for the much stronger phonon softening in crystalline GeTe.

A film‐averaged heating estimate yields temperature rises of only a few kelvin at 1 mJ cm^−2^, suggesting that heating alone cannot explain the much stronger phonon softening in crystalline GeTe (see Supporting Information).

Overall, the fluence‐dependent measurements reveal a strong lattice response in crystalline GeTe, characterized by pronounced phonon softening and amplitude growth, whereas the coherent phonons in GeSe remain comparatively weak and show only minor fluence‐dependent changes.

## 3 Discussion and Conclusion

The markedly different ultrafast lattice dynamics observed in GeTe and GeSe can be understood in terms of their underlying chemical bonding. In conventional covalent glasses such as SiO_2_, GeSe_2_, and GeSe, the chemical bonding in the amorphous and crystalline phases is very similar, resulting in comparable short‑range order and related physical properties [8, 43]. In contrast, several phase‑change materials including GeTe exhibit a fundamentally different behavior: their crystalline phases employ metavalent bonding, whereas the amorphous phases are covalently bonded [8, 16]. This change in bonding is accompanied by pronounced differences in atomic arrangement and opto‑electronic properties between the glassy and crystalline states. Within this framework, GeTe can therefore be classified as a non‑Zachariasen glass, while GeSe represents a conventional covalent glass where the bonding remains largely unchanged upon crystallization [8].

This distinction between GeTe and GeSe is also supported by atom probe tomography (APT) measurements revealing their bond rupture characteristics. Figure 5 shows the probability of molecular ions (PMI) plotted against the probability of multiple events (PME) for the amorphous and crystalline phases of both materials. These quantities characterize the bond rupture during laser‐assisted field evaporation and have been shown to distinguish different bonding mechanisms in solids [9, 16]. Recent APT studies of bulk chalcogenides and phase‐change alloys provide further context for interpreting these bond‐rupture characteristics [44].

**FIGURE 5:**
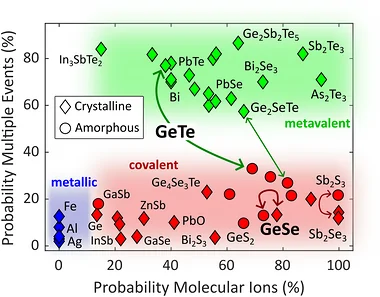
Bond‐rupture characteristics obtained from atom probe tomography. The probability of multiple events (PME) is plotted against the probability of molecular ions (PMI) for amorphous and crystalline solids, including GeTe and GeSe films. Crystalline GeTe exhibits a strong increase in PME compared to its amorphous counterpart, consistent with a transition from covalent to metavalent bonding. In contrast, amorphous and crystalline GeSe display nearly identical PMI and PME values, indicating similar covalent bonding in both phases. Adapted with permission from Ref. [9]. Copyright 2024, Wiley‐VCH GmbH. Additional literature data from Refs. [16, 46] and data points for the GeTe and GeSe films measured in this work are included.

For GeTe, crystallization leads to a pronounced increase in PME from about 33% in the amorphous state to 77% in the crystalline phase, accompanied by a reduction in PMI from 69% to 38%. Such a high PME value is characteristic of metavalent bonding and reflects enhanced correlated evaporation processes [9, 45]. In contrast, amorphous and crystalline GeSe exhibit very similar PMI and PME values, indicating that the bonding character remains largely unchanged upon crystallization. This behavior is consistent with the classification of GeSe as a conventional covalent glass and with the behavior seen in covalent low optical loss PCM Sb_2_S_3_ [46]. Evaporation conditions and further APT data is shown in the supplementary.

The ultrafast response of crystalline GeTe reflects the particular sensitivity of metavalently bonded systems to electronic excitation [42]. In our measurements, crystalline GeTe exhibits strong coherent phonon oscillations together with pronounced fluence‑dependent phonon softening and a significant increase in oscillation amplitude. In particular, the pronounced frequency softening indicates a substantial photoinduced change in the effective force constant and p‐orbital overlap, whereas the measured oscillation amplitude also depends on how efficiently lattice motion modulates the probe reflectivity. Such behavior is consistent with the highly polarizable bonding network of metavalent materials, in which small perturbations of the electronic system can strongly affect the lattice potential [15]. The Peierls‐like distortion identifies an *A*_1_ coordinate that changes the asymmetry between short and long Ge‐Te bonds, while metavalent bonding describes the polarizable electronic environment whose forces are particularly sensitive to changes in occupation along this coordinate [21]. Under femtosecond excitation, the sudden change in electronic population can therefore shift the equilibrium atomic positions and efficiently launch coherent lattice motion via the displacive excitation of coherent phonons mechanism [41]. The oscillatory reflectivity signal depends on the displacement derivative of the dielectric tensor ∂*ε*_*ij*_(*ω*_probe_)/∂*Q* which is related to the Raman tensor and governs the optical sensitivity to the *A*_1_ motion. Consequently, the increase of the measured amplitude reflects both more efficient generation of coherent displacement and stronger optical transduction.

Another notable feature of crystalline GeTe is the unusually short coherent phonon dephasing time extracted from the pump‐probe measurements. The dephasing time in the crystalline phase is shorter than in the amorphous films despite the higher structural order of the crystal. This observation is remarkable because phonon damping in disordered materials is typically enhanced by structural disorder and localization of vibrational modes. In the present case, however, the crystalline phase exhibits even stronger damping than the amorphous state. This suggests that intrinsic anharmonicity of the lattice potential contributes substantially to the rapid coherent‐phonon decay in crystalline GeTe and may outweigh disorder‐induced dephasing in the amorphous phase. Such strong anharmonicity is consistent with the bonding characteristics of crystalline GeTe, where the competition between electron localization and delocalization produces a shallow and highly anharmonic potential landscape [21]. A similar contrast between more anharmonic crystalline and more nearly parabolic amorphous potentials has been reported for other PCMs [47].

In contrast to GeTe, the coherent phonon response of GeSe is significantly weaker. In crystalline GeSe only small oscillatory contributions are observed, while no coherent oscillations are detected in the amorphous phase. This behavior is consistent with the Raman spectra, where amorphous GeSe exhibits only weak and strongly broadened vibrational features without well‐defined peaks. In addition, the coherent phonon frequencies in crystalline GeSe show only weak fluence‐dependent shifts, indicating a comparatively small excitation‐induced modification of the lattice potential.

The pronounced photoinduced softening observed in Bi provides a useful parallel to GeTe: both materials are classified as metavalently bonded and exhibit a strong response of a Peierls‐sensitive mode to electronic excitation [27, 48]. Crystalline GeSe has also been described as Peierls‐distorted, but with a larger distortion amplitude than GeTe [49]. Nevertheless, its property fingerprint supports predominantly covalent bonding, and its coherent phonons exhibit much weaker fluence‐dependent softening. As discussed for related chalcogenides [49], the magnitude of the distortion is relevant: a moderate distortion can retain substantial electronic delocalization, whereas a stronger distortion favors localization and covalent bonding. Thus, the presence of a Peierls‐like distortion alone does not determine either the bonding character or the magnitude of the photoinduced phonon response [49].

The low oscillation amplitudes and small fluence‐induced frequency shifts observed in GeSe are consistent with its covalent bonding character [42]. In covalently bonded systems, the interatomic potential is less susceptible to perturbations of the electronic system, resulting in a smaller driving force for coherent lattice motion. A similar behavior has been observed in covalently bonded In_2_Te_3_ thin films, where the coherent phonon frequencies remain essentially unchanged with increasing excitation fluence [50]. This contrasts with the pronounced nonlinear lattice response observed in metavalently bonded GeTe.

It should be noted that the term “coherent phonon” is formally defined for crystalline solids where lattice periodicity allows phonon modes to be described by well‐defined wave vectors. In amorphous materials, translational symmetry is absent, and vibrational excitations are more appropriately described as localized or quasi‐localized modes. Nevertheless, ultrafast pump‐probe experiments can still drive a large number of similar local oscillators into a synchronized motion, resulting in a measurable oscillatory signal in the transient reflectivity. In this sense, the oscillations observed in amorphous GeTe can be interpreted as coherently excited vibrational modes associated with characteristic local structural motifs. The absence of such oscillations in amorphous GeSe indicates that the vibrational modes in this material cannot be efficiently driven into a collective coherent motion under the present optical excitation conditions, consistent with the weak and strongly broadened Raman features observed for this state.

Finally, the comparison between the as‑deposited and relaxed amorphous states shows that annealing below the glass transition temperature does not significantly influence the coherent phonon dynamics in either GeTe or GeSe. While such relaxation processes can modify subtle aspects of the amorphous structure, the ultrafast lattice response remains essentially unchanged. This observation further supports the view that the coherent phonon dynamics in these materials are primarily governed by the underlying bonding mechanism rather than by minor variations in the local amorphous structure.

Taken together, the results demonstrate that coherent phonon spectroscopy provides a sensitive probe of bonding mechanisms in chalcogenide materials. Systems with metavalent bonding, such as crystalline GeTe, exhibit strong and highly nonlinear lattice responses to optical excitation, whereas covalently bonded systems like GeSe display much weaker and largely fluence‑independent phonon dynamics.

## 4 Methods

### 4.1 Sample Preparation

GeTe and GeSe thin films were deposited by magnetron sputtering from stoichiometric targets onto single‑side polished Si substrates. The base pressure of the deposition chamber was 3 × 10^−6^ mbar. The films were deposited in the amorphous state with a thickness of approximately 300 nm. To prevent oxidation and increase the optical damage threshold, the films were capped in situ with a 20 nm thick ZnS_80_:(SiO_2_)_20_ layer deposited by RF magnetron sputtering.

Crystalline samples were obtained by annealing the as‑deposited films in a tube furnace under an inert atmosphere. GeTe films were annealed at 300°C for 15 min using a heating rate of 2 K min^−1^, while GeSe films were annealed at 350°C for 15 min with a heating rate of 1 K min^−1^. To obtain relaxed amorphous states, additional annealing was performed below the glass transition temperature for 1 h and 24 h at 145°C for GeTe and 265°C for GeSe.

### 4.2 Grazing‑Incidence X‑Ray Diffraction

Structural characterization was performed using grazing‐incidence X‐ray diffraction (GIXRD) on a Bruker D8 Discover diffractometer equipped with a Cu K_α_ radiation source (mean value λ = 1.5418 Å) and a LynxEye detector operated in 0D mode. The measurements were performed in parallel‐beam geometry using a Göbel mirror, resulting in illumination with the Cu K_α_ doublet. Diffraction patterns were recorded at a fixed incident angle of 1° with a step size of 0.025° and an integration time of 3 s per step. Peak positions were indexed to rhombohedral GeTe (ICSD 101817) and orthorhombic GeSe (ICSD 230429) [36, 37].

### 4.3 Raman Spectroscopy

Raman spectra were measured using a commercial WITec alpha300 system in backscattering geometry. Excitation was provided by a diode‐pumped solid‐state laser with a wavelength of 531 nm (2.33 eV). The laser beam was focused onto the sample using a 50× objective (NA = 0.7), resulting in a spot size of approximately 400–500 nm. For the crystalline samples, the excitation power was limited to 500 µW to avoid laser‐induced heating. For the amorphous samples, the maximum excitation power was set by assuring no crystallization during the measurements. For this after each measurement, the spots were controlled with the optical microscope. The maximum power where no crystalline spots were visible was used for the recorded spectra. This power was about 100 µW. The scattered light was analyzed using a spectrometer equipped with a 2400 lines mm^−1^ grating and detected by a charge‐coupled device detector. All measurements were performed at room temperature.

### 4.4 Ultrafast Pump Probe Reflectivity

Ultrafast optical measurements were performed using a standard two‐color pump‐probe setup in reflection geometry. The pump pulses (800 nm wavelength, 60 fs duration) were generated by a Ti: sapphire regenerative amplifier and modulated using a mechanical chopper at 1500 Hz. The pump beam was directed through a motorized delay line and focused onto the sample to a spot diameter of approximately 150 µm.

The probe pulses were generated by frequency conversion in an optical parametric amplifier, yielding a wavelength of 516 nm. The probe beam was focused to a spot size of approximately 30 µm. The reflected probe signal was detected using balanced Si photodiodes connected to variable‐gain current amplifiers and recorded using a data acquisition card. The pump fluence was varied between 0.2 and 3 mJ cm^−2^, while the probe fluence was kept at least one order of magnitude lower. Transient reflectivity changes ΔR/R were obtained by normalizing the pump‐induced reflectivity change to the steady‐state reflectivity R_0_. To minimize systematic errors, the order of delay positions during data acquisition was randomized.

### 4.5 Atom Probe Tomography

Atom probe tomography (APT) measurements were performed to investigate bond‐rupture characteristics of the films. Needle‐shaped specimens were prepared using a dual‐beam focused ion beam (FIB) system (Helios NanoLab 650, FEI) following the standard lift‐out procedure. APT experiments were carried out using a commercial CAMECA LEAP 5000XS instrument operated in laser‐assisted mode with a laser wavelength of 355 nm. The laser pulse energy was 15–20 pJ, the pulse repetition rate was 200 kHz for GeSe and 50 kHz for GeTe, and the specimen temperature was maintained at 40 K. The average detection rate was 1.0%. Data reconstruction was performed using APSuite 6.3 software and the EPOSA analysis package. From the reconstructed datasets, the probability of molecular ions (PMI) and the probability of multiple events (PME) were extracted to characterize bond rupture during field evaporation.

## Author Contributions

T. Veslin and F. Hoff contributed equally to the project. F. Hoff initiated the project. M. Wuttig and P. Lucas supervised the project. T. Veslin performed pump‐probe and Raman measurements. F. Hoff analyzed the data. F. Nöhl performed XRD measurements. J. Köttgen and E. Hildebrand performed APT measurements and analyzed the data. F. Hoff prepared the figures. T. Veslin and F. Hoff wrote the initial manuscript. M. Wuttig and P. Lucas revised the manuscript.

## Funding

Federal Ministry of Research, Technology and Space BMFTR (03ZU2106BA), National Science Foundation NSF‐DMR (#1832817).

## Conflicts of Interest

The authors declare no conflicts of interest.

## Supporting information




**Supporting File**: smll76481‐sup‐0001‐SuppMat.docx.

## Data Availability

The data that support the findings of this study are available from the corresponding author upon reasonable request.
