# Supplementary material for: Ultrafast Coherent Phonon Dynamics in GeTe and GeSe: Contrasting Metavalent and Covalent Bonding
Source: Small. 2026 Oct 27;22(63):e76481. doi: 10.1002/smll.76481 (PMC13755996; doi:10.1002/smll.76481)
Supplement: Supplementary file 1 [file SMLL-22-e76481-s001.docx]

**Supporting Information**

**Ultrafast Coherent Phonon Dynamics in GeTe and GeSe: Contrasting Metavalent and Covalent Bonding**

Timo Veslin, Felix Hoff*, Felix Nöhl, Elias Hildebrand, Jan Köttgen, Pierre Lucas, Matthias Wuttig

**T. Veslin, F. Hoff, F. Nöhl, E. Hildebrand, J. Köttgen, and M. Wuttig**

Institute of Physics IA, RWTH Aachen University, 52074 Aachen, Germany

E-Mail: hoff@physik.rwth-aachen.de

**P. Lucas**

Department of Materials Science and Engineering, University of Arizona, Tucson, Arizona 85721, USA

**M. Wuttig**

Peter-Grünberg-Institute – JARA-Institute Energy Eﬃcient Information Technology (PGI-10) Wilhelm-Johnen-Straße, 52428 Jülich, Germany

**Keywords**:

coherent phonons, metavalent bonding, phase change materials, anharmonicity, chalcogenides, unconventional glasses

The oscillatory components of the transient reflectivity signals shown in Fig. 3 of the main text were analyzed using damped harmonic oscillator fits. To obtain the oscillatory signals, the electronic background was first fitted and subtracted from the measured transient reflectivity traces. The resulting oscillatory components were then fitted using a damped harmonic oscillator model to extract the coherent phonon frequencies and dephasing times. Figure S1 shows representative fits for the as‑deposited amorphous, relaxed amorphous, and crystalline GeTe films as well as for crystalline GeSe. The extracted parameters and their uncertainties obtained from the fitting procedure are indicated in the individual panels.

Figure S1: Damped harmonic oscillator fits of the oscillatory components extracted from the transient reflectivity traces shown in Fig. 3 of the main text. The oscillatory signals obtained after subtraction of the electronic background are shown in blue, while the corresponding fits are shown in red. (a) As‑deposited amorphous GeTe, (b) relaxed amorphous GeTe, (c) crystalline GeTe, and (d) crystalline GeSe. The fitted coherent phonon frequencies and dephasing times are indicated in each panel together with the corresponding 95% confidence bounds obtained from the fitting procedure.

To determine the phonon frequencies indicated in Fig. 2 of the main text, the Raman spectra of the crystalline films were fitted using Lorentzian line shapes. The corresponding fits are shown in Fig. S2.

Figure S2: Lorentzian peak fits of the Raman spectra of the crystalline (a) GeTe and (b) GeSe films. Background subtracted data is shown in black and the total fit (as a sum of several peaks) in red. The extracted peak positions correspond to the dashed lines indicating the phonon frequencies in Fig. 2 of the main text.

To illustrate the fluence dependence discussed in Fig. 4 of the main text, the transient reflectivity traces used for the analysis are shown in Figures S2 and S3. The figures display the raw pump-probe signals measured at all investigated pump fluences for the different structural states of GeTe and GeSe. These datasets form the basis for the damped harmonic oscillator fits from which the coherent phonon frequencies, oscillation amplitudes, and dephasing times presented in Fig. 4 were extracted.

Figure S3: Transient reflectivity traces ΔR/R of GeTe thin films measured at different pump fluences. Panels show the responses for (a) as‑deposited amorphous GeTe, (b,c) relaxed amorphous GeTe, (d) crystalline GeTe. The measurements correspond to the raw data used to extract the coherent phonon parameters summarized in Fig. 4 of the main manuscript.

Figure S4: Transient reflectivity traces ΔR/R of GeSe thin films measured at different pump fluences. Panels show the responses for (a) as‑deposited amorphous GeSe, (b,c) relaxed amorphous GeSe, (d) crystalline GeTe. The measurements in (d) correspond to the raw data used to extract the coherent phonon parameters summarized in Fig. 4 of the main manuscript.

For the multiplicity analysis of amorphous and crystalline GeSe and GeTe, the evaporation conditions were evaluated using correlation histograms, which allow assessment of the quality of the atom probe acquisition. [10.1016/j.ultramic.2010.11.021] All histograms exhibit strongly correlated evaporation without signatures of DC‑evaporation or heat tails. In the case of crystalline GeSe a small amount of molecular ion dissociation is observed.

The 3D multiplicity analysis reveals a clear distinction between crystalline GeTe and the other samples. Crystalline GeTe exhibits a significantly higher probability of multiple events (PME), whereas the covalently bonded samples (amorphous GeTe and both GeSe phases) display much lower PME values of approximately 13–33%.

For the GeSe samples, APT measurements were performed using a laser pulse energy of 20 pJ and a repetition rate of 200 kHz at a base temperature of 40 K. For GeTe, a reduced repetition rate of 50 kHz and a base temperature of 30 K were used to avoid laser‑induced crystallization of the tip during acquisition, as previously reported for the related compound Ge_2_Sb_2_Te_5_. [10.1002/adma.201706735] Indeed, a laser pulse energy of 20 pJ resulted in in‑situ crystallization of an amorphous GeTe tip, as shown in Fig. S6. Stable evaporation of the amorphous GeTe samples was achieved by reducing the laser pulse energy to 15 pJ.

For laser energies below 15 pJ the PME values remained low, confirming that the amorphous phase was preserved during the measurement. The PME and PMI values obtained for the in‑situ crystallized tip agree well with previously reported values for bulk crystalline GeTe [10.1002/adma.201706735] and with the APT measurements of thermally crystallized GeTe thin films shown in Fig. S7.

Figure S5: Correlation histograms of amorphous and crystalline GeTe and GeSe. The evaporation events are strongly correlated for all samples.

Figure S6: 3D multiplicity distributions of all four samples showing the pronounced difference in bond‑rupture behavior for crystalline GeTe.

Figure S7: Comparison of in‑situ laser‑crystallized GeTe and thermally crystallized GeTe, showing no significant differences in the atom probe measurements.

To clarify the relative Raman intensities of the different structural states, Figure S8 shows unnormalized Raman spectra. The normalized spectra in Fig. 2 of the main text emphasize spectral shape and mode positions, whereas the raw spectra demonstrate the much weaker Raman response of the amorphous films, compared to the corresponding crystalline phases.

Figure S8: Unnormalized Raman spectra of (a) GeTe and (b) GeSe thin films in the as-deposited amorphous, relaxed amorphous, and crystalline states. In contrast to the normalized spectra shown in Fig. 2 of the main text, the raw spectra show that the Raman response of amorphous GeSe is much weaker than that of crystalline GeSe.

Estimation of pump-induced temperature increase

Assuming that the absorbed energy is initially deposited in the film and is converted into lattice heat after carrier-lattice equilibration (ns-µs time scale), the film-averaged temperature increase is estimated from

$$\Delta T=\frac{F_{\text{abs}}}{\rho C_{p}d}=\frac{FA}{\rho C_{p}d},$$

where $F$ is the incident fluence, $A$ is the absorbed fraction including the finite film thickness, $\rho$ is the mass density, $C_{p}$ is the specific heat capacity, and $d$ is the film thickness. This estimate neglects heat diffusion into the substrate and therefore represents the initial temperature increase of the film.

For crystalline GeSe, we used $F=1 \text{mJ c}\text{m}^{\text{-2}}$, $A_{\text{GeSe}}=0.17,\rho_{\text{GeSe}}=5.6 \text{g c}\text{m}^{\text{-3}}, C_{p,\text{GeSe}}=350 \text{mJ }\text{g}^{\text{-1}}\text{ }\text{K}^{\text{-1}},d=300 \text{nm}:$

$$\Delta T_{\text{GeSe}}=\frac{1.70}{0.588}\approx2.9 \text{K}.$$

For crystalline GeTe, we used $F=1 \text{mJ c}\text{m}^{\text{-2}}$, $A_{\text{GeTe}}=0.38,\rho_{\text{GeTe}}=6.14 \text{g c}\text{m}^{\text{-3}}, C_{p,\text{GeTe}}=250 \text{mJ }\text{g}^{\text{-1}}\text{ }\text{K}^{\text{-1}},d=300 \text{nm}:$

$$\Delta T_{\text{GeTe}}=\frac{3.78}{0.461}\approx8.2 \text{K}.$$

These estimates indicate that the lattice-heating contribution at $1 \text{mJ c}\text{m}^{\text{-2}}$ is on the order of a few kelvin in GeSe and approximately $8 \text{K}$ in GeTe. Heat transfer into the substrate will reduce the temperature at later delays, while the initial carrier and lattice temperatures may not be in equilibrium during the earliest part of the transient response.

Ellipsometry and estimation of absorbed pump fluence.

Spectroscopic ellipsometry was performed on comparable PCM reference films with thicknesses on the order of 100 nm and approximately 20 nm capping layers. Spectra were acquired at angles of incidence of 65°, 70°, and 75° using a J.A. Woollam M-2000UI ellipsometer over a photon-energy range of 0.72–5.14 eV. The film dielectric functions were extracted in CompleteEASE using a capping-layer/PCM-film/substrate optical model. The thicknesses of the reference films were determined by X-ray reflectivity and used in the ellipsometric analysis.

The resulting dielectric-function are shown in Figure S9. To estimate the fraction of incident pump fluence absorbed by the films used for the pump-probe measurements, we used their nominal thickness d=300 nm and calculated the absorbed fraction as written in the main text.

Figure S9: Spectroscopic dielectric functions of amorphous (a-) and crystalline (c-) GeTe and GeSe reference films. (a) Real part $\varepsilon_{1}$ and (b) imaginary part $\varepsilon_{2}$ for a-GeTe and c-GeTe. (c) Real part $\varepsilon_{1}$ and (d) imaginary part $\varepsilon_{2}$ for a-GeSe and c-GeSe.
